# Stochastic resonance stimulation effect on stability during walking in people with Parkinson disease

**DOI:** 10.1080/23335432.2025.2584665

**Published:** 2025-11-06

**Authors:** Eman Alsaqabi, Stephen DiBianca, Ashwini Sansare, Khushboo Verma, Hendrik Reimann, John Jeka

**Affiliations:** a Department of Physical Therapy, College of Allied Health Sciences, Kuwait University, Kuwait City, Kuwait; b Department of Kinesiology and Applied Physiology, University of Delaware, Newark, DE, USA; c Department of Kinesiology and Sports Medicine, Texas A&M University, College Station, TX, USA; d Department of Physical Therapy, University of Delaware, Newark, DE

**Keywords:** Electric stimulation therapy, gait, Parkinson disease, postural control, somatosensory deficits, stochastic resonance

## Abstract

People with Parkinson disease (PwPD) often face challenges with maintaining balance while walking, which can stem from sensory dysfunction. Studies have identified different biomechanical strategies that aid in preserving upright balance control. Stochastic resonance (SR) stimulation delivers sub-threshold electrical noise to enhance the detection capabilities of dysfunctional sensory systems. Yet, the effectiveness of SR in enhancing gait stability in PwPD is undetermined. The purpose of this study was to investigate the effects of SR on balance control during visually perturbed walking in PwPD (NCT06829342).

Fourteen individuals with PD completed the study. We established individualized sensory thresholds for SR stimulation and identified the optimal SR intensity. Following this, the participants walked within a virtually perturbed environment. Center of mass (CoM) excursion, foot placement, and ankle roll responses were assessed bilaterally.

Peak CoM excursion showed a significant increase, indicating reduced stability, with the SR condition compared to no-SR at the more affected side. Outcome measures related to balance control mechanisms were insignificant.

With SR, PwPD were driven by the induced fall with more sway and without significant alterations in balance strategies, which might be due to adding more noise to sensory processing and misidentifying the more affected side.

## 1. Introduction

Balance impairment is a significant concern for people with Parkinson disease (PwPD), which degrades as the disease progresses (Ehgoetz Martens et al. 2013; Bernardinis et al. 2021; Halperin et al. 2021; Kelemen et al. 2022; Corrà et al. 2023). PwPD who have balance problems have a lower quality of life and less functional independence, according to research that indicated lower scores in physical activity, social capacity, and general health assessments (Navarro-Flores et al. 2022). Contributing factors impact postural stability in this population range from central factors such as processing abilities impairments to more peripheral ones such as muscle weakness (Hwang et al. 2016; Gamborg et al. 2023; Bath and Wang 2024). Fear of falling and kinesiophobia worsen these problems as found in 77.3% of PwPD to have moderate to severe kinesiophobia compared to healthy controls, creating a cycle of less activity and worse mobility that goes beyond motor symptoms (Jiménez-Cebrián et al. 2021). While primary motor symptoms of tremors, bradykinesia, and rigidity receive most attention, instability may also stem from sensory impairments (Conte et al. 2013; Ehgoetz Martens et al. 2013; Bernardinis et al. 2021; Halperin et al. 2021; Kelemen et al. 2022; Corrà et al. 2023). Maintaining upright stability requires integrating sensory information from visual, vestibular, and somatosensory systems (Xie et al. 2017; Zarkou et al. 2018; Roytman et al. 2025). Studies reported proprioceptive and vibrotactile deficits in the PD population, which could relate to postural instabilities (Elangovan et al. 2018; Heß et al. 2023; Roytman et al. 2025). This relation manifests through impaired quality of sensory information conveyed to the central nervous system through afferent sensory neurons during environmental changes (Roytman et al. 2025). Gorst and colleagues emphasized ankle proprioception and foot sole vibrotactile sensitivity deficits contributing to balance issues in PwPD (Gorst et al. 2019). A recent study found that PwPD poorly perceive vibration on foot soles compared to a healthy population, leading to faulty motor performance. Their findings underscore the critical role of lower extremity somatosensory processing in maintaining movement and stability (Heß et al. 2023). Impairments in visual, vestibular, or somatosensory systems might impact proper integration and alter reliance on each system in different contexts.

Given these sensory function consequences, researchers are investigating new interventional methodologies. Stochastic resonance (SR) stimulation shows promise in improving sensory functions and mitigates disabilities (White et al. 2019; Zandiyeh et al. 2019). SR uses subthreshold noise to amplify weak signals to a detectable threshold, enhancing sensation sensitivity (White et al. 2019; Sansare A, Arcodia M, et al. 2024). Its effect extends beyond skin receptors to receptors in muscles, tendons, and joints (White et al. 2019). SR effectiveness has been demonstrated in improving somatosensory acuity after knee surgery and enhancing postural control in children with cerebral palsy (Zarkou et al. 2018; Zandiyeh et al. 2019; Sansare A, Arcodia M, et al. 2024). While these suggest potential value in enhancing proprioceptive function and balance, few studies have focused on SR’s specific role in PD.

Upright stability results from strategies involving center of mass (CoM) and center of pressure interaction, activating sensorimotor networks and coordinated lower-limb muscle contractions (Reimann et al. 2017, 2019). Three critical balance strategies are foot placement, ankle roll, and push-off by proximal and distal joints (Reimann et al. 2017, 2019). While hip joints activate during foot displacement, ankle-roll and push-off mechanisms involve foot and ankle joints and musculatures (Reimann et al. 2017). Both distal and proximal strategies are important, but distal mechanisms predominantly manage walking cycle, ground adaptation, and metabolic optimization (Rinalduzzi et al. 2015; Reimann et al. 2017, 2019; Islam et al. 2020). During walking, our CoM constantly moves while balance mechanisms vary center of pressure to maintain stability (Reimann et al. 2017, 2019).

PwPD show atypical gait and altered balance mechanisms, engaging proximal joints more than distal ones compared to healthy individuals (Cioni et al. 1997; Arcodia et al. 2020; Islam et al. 2020). This proximal over-distal activation could result from defective sensory input from distal joints, impairing ability to distinguish surface conditions (Gorst et al. 2019). Arcodia’s study showed older people with and without PD recruit balance mechanisms throughout visually perturbed walking, though with different variability and onsets (Arcodia 2024). Results indicated more varied muscle activities and subtalar joint angles in healthy older population (Arcodia 2024). Larger steps toward perceived fall direction resulted in earlier gluteus medius activation in healthy population than in PwPD. Increasing base of support allowed more flexible CoM movement before stability loss (Arcodia 2024). The primary aim of this study was to investigate how customized SR stimulation affects the medio-lateral CoM excursion and balance mechanisms immediately when the PD participants walk while their vision is disturbed. We hypothesized that SR stimulation would make the lateral body sway less and improve distal balance techniques in corresponding body sway changes compared to the no stimulation condition. These alterations would enhance dynamic stability in the presence of visual disturbances.

## 2. Materials and methods

### 2.1. Participants

Fifteen participants, who were clinically diagnosed with Parkinson disease, Hoehn and Yahr stages (H&Y) ≤ III, were recruited. Initial screening for eligibility was performed via phone or email based on the inclusion and exclusion criteria listed in Table 1. In addition to the listed exclusion criteria, PD participants were going to be excluded if they exhibited severe dyskinesia, did not respond to L-DOPA or other dopaminergic treatment, or exhibited cardiovascular-autonomic or multiple-system symptoms indicative of a ‘Parkinsonism-plus’ presentation. Once eligibility was established, an informed consent form was obtained. The data was analyzed based on laterality. We identified the more affected side in Parkinson Disease as one of the participants reported experiencing dominant motor symptoms and confirmed by the MDS-UPDRS-III results (primarily tremors in the upper extremity), leaving the opposite side as the less affected side. The University of Delaware Institutional Review Board approved the study protocol. This study is retrospectively registered in ClinicalTrials.gov (NCT06829342).

**Table 1.** Inclusion and exclusion criteria.

Inclusion	Exclusion
Age 40 – 85 years old Clinical diagnosis of Parkinson Disease Hoehn and Yahr (H&Y) stage ≤ III Can walk independently for at least 2 minutes without an assistive device or orthosis Ability to communicate discomfort during testing Can follow multi-step commands Scored at least 24/30 on the Montreal Cognitive assessment (MoCA)	Any head, neck, or face injury in the six months prior to the study (e.g. concussion, eye injury) History of vestibular or ocular dysfunction Currently taking any medications affecting balance (i.e. antibiotics) Injuries to lower extremities affecting balance in the past six months Pregnancy Any neurological disorders other than Parkinson’s disease (e.g. seizure disorders, closed head injuries with loss of consciousness greater than 15 minutes, CNS neoplasm, history of stroke) Unstable cardiac or pulmonary disease Clinically obese (BMI 30 or above) Any metal implant in the feet or legs that is close to the stimulating electrodes A known allergy to medical-grade adhesives Any other comorbidity affecting the ability to safely walk without assistance for at least 2 minutes

### 2.2. Instrumentation

Participants participated in a walking paradigm on a split-belt treadmill (Bertec Inc., Columbus, Ohio, USA) set within an immersive virtual reality screen that displayed a 4-meter-wide corridor surrounded by floating cubes, created using Unity3D software from Unity Technologies in San Francisco, CA, USA. The treadmill was self-paced, where the belt’s speed adjusted to match the walking pace chosen by the participant through a custom program developed in Labview (National Instruments Inc., Austin, TX, USA). To ensure safety, participants were always secured with a harness to prevent falls. The experimental setup included the use of a 13-camera motion capture system by Qualysis Inc., from Gothenberg, Sweden, using 44 reflective markers for capturing full-body movement data (Davis et al. 1991). Data collection was conducted at a frequency of 200 Hz for marker data and 1000 Hz for recording ground reaction forces and moments. The analysis of ground reaction data was refined through a low pass filter, specifically a 4^th^ order Butterworth filter with a threshold of 20 Hz. Utilizing custom MATLAB scripts, we analyzed the data to calculate the outcome measures outlined in the Outcome Measures section.

**Figure 1.**
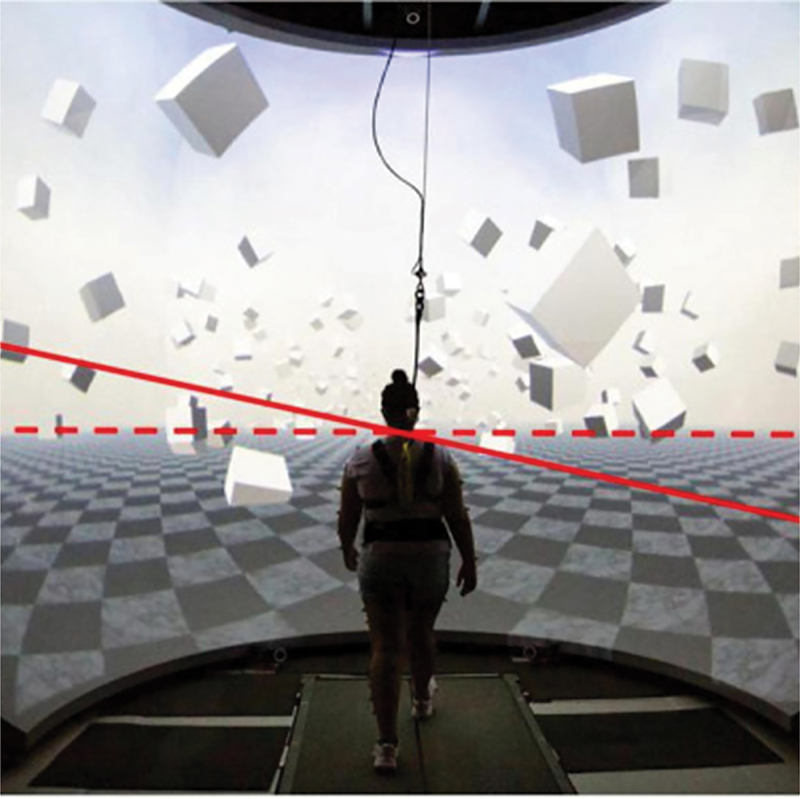
Virtual environment setup and virtual perturbation, with the red dashed line representing the horizon and the solid line representing its tilting direction. Figure adapted from Sansare et al. (2022).

Figure 1 illustrates the virtual reality cave and the scene employed in our study protocol, offering an engaging VR experience akin to real-world over-ground walking. In this protocol, visual perturbation was administered to help understand whether enhancing distal somatosensory inputs would lead to better stability by upweighting proprioceptive signals while downweighing visual inputs, which becomes less reliable. To introduce visual disturbances, the scene rotated about the treadmill’s central anterior-posterior axis. This rotation involved an angular acceleration of 45 degrees per second squared for 600 milliseconds upon the heel strike of either foot. The scene then maintained its inclined position for 2,000 milliseconds before returning to a level position over an additional 1,000 milliseconds at a steady angular speed. This visual perturbation mimics the sensation of a sideways fall. The disturbances occurred at pseudo-randomly heel strikes of either foot, with each event followed by a washout phase of 10 to 12 steps to allow the visual environment to stabilize before the next disturbance. To assess the impact of these visual perturbations on walking, we interspersed actual visual stimuli with sham stimuli periods, during which participants walked without disturbances for 10 to 12 steps, allowing for uninterrupted walking.

### 2.3. Stochastic resonance

**Figure 2.**
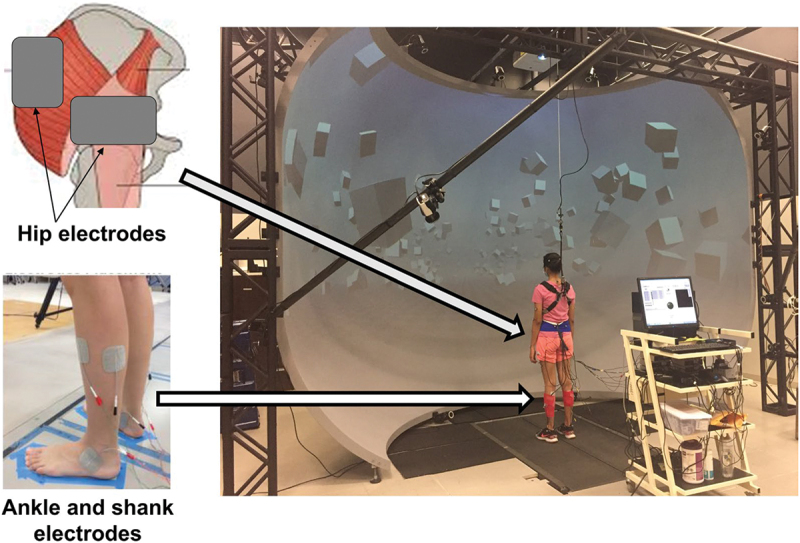
The experimental setup included the motion capture system, self-paced treadmill, and the sr stimulation cart. The cart had a computer responsible for generating the sr signal, and six stimulators that administered electric stimulation through surface electrodes placed on the hip, shank, and ankle. Figure adapted from Sansare et al. (2024).

A specialized Labview application produced the elecrical SR signal, characterized by uniform white noise, to activate six stimulators (STMISOLA, Biopac Systems, Inc., Goleta, USA). The intensity of the SR is defined by the amplitude of the white noise signal. We affixed self-adhesive electrodes 1) on the ankle at the anterior talofibular and deltoid ligaments, 2) on the shank covering the lateral soleus, peroneus longus, and tibialis anterior muscles, and 3) around the hip area, specifically inferior and posterolateral to the greater trochanter, targeting the hip joint capsule as well as the gluteus medius and maximus muscles. Figure 2 represents the experimental setup.

### 2.4. Protocol

Participants underwent a minimum of two practice sessions lasting 2 minutes each. The first session involved walking on the self-paced treadmill without visual disturbances, allowing them to acclimate to the self-paced treadmill. In the second session, they experienced the treadmill with visual perturbations to familiarize themselves with the virtual environment and the induced visual disturbances. Additional practice trials were offered if needed. The experimental protocol was adapted from Sansare and colleagues (Sansare A, Arcodia M, et al. 2024) and was structured as follows:

1. Establish individual SR sensory threshold: We set the baseline for SR stimulation intensity by assessing each participant’s sensory detection threshold at each stimulation site. This threshold was the lowest level of stimulation at which an individual perceived a slight tingling. During the assessment, participants walked on the treadmill at their chosen steady speed. We then gradually increased the stimulation by 0.1 mA increments until the participants acknowledged the tingling sensation. To confirm this threshold, the intensity was reduced until the sensation was no longer perceptible. This process was conducted three times, and the sensory threshold for each site was determined as the minimum intensity at which stimulation was felt across these trials.
2. Identifying SR Optimal Intensity: We determined optimal intensity for each participant by evaluating their balance performance at 25%, 50%, 75%, and 90% of their specific sensory threshold identified previously. Participants underwent 2-minute walking sessions on the treadmill at these intensities, with rest periods between sessions. The order of the SR intensities was randomized using a computer protocol. Balance was quantified using the minimum lateral margin of stability. A higher margin of stability indicates that a greater force is needed to cause instability, reflecting better balance. The SR intensity level that provided the best protection against lateral falls, indicated by the largest increase in margin of stability, was considered the optimal SR intensity for each participant and was selected for further tests. All SR intensity levels were sub-threshold, meaning they did not exceed 100% of the sensory threshold, ensuring that participants were unaware of the stimulation intensity throughout the trials.
3. Visual Perturbation Method: To explore how SR stimulation impacts balance, participants were subjected to SR stimulation at the optimal intensity and to a no stimulation (no-SR) scenario as a baseline comparison. They undertook three two-minute trials under both conditions (SR and no-SR), during which they experienced the aforementioned visual perturbations. The order of the SR and no-SR conditions was randomized, and participants were not informed of the condition they were undergoing at any given time.

### 2.5. Outcome measures

To evaluate the impact of SR on the body’s reaction to visual disturbances, we primarily focused on the area under the curve (AUC) of the medio-lateral (ML) CoM displacement. This was determined by comparing the average CoM during perturbed steps to non-perturbed steps for each participant, integrated across the first eight steps initiated by the heel strike that triggered the stimulus. During this interval, we assessed the magnitude of CoM displacement by identifying the maximum excursion point, or Peak ML CoM excursion. The timing of the CoM’s response was gauged by measuring the interval from the perturbation’s start to the peak of the CoM movement, or Peak Time. We included the ankle’s rotational movement, ankle roll, and foot positioning strategies, foot placement, as our secondary outcome measures. The ankle’s rotation, or subtalar angle, was measured during the initial single stance phase post-perturbation and integrated to produce the AUC of the subtalar angle. The foot placement response was analyzed based on the distance between the lead foot relative to the back foot in the medial-lateral direction at heel strike. The mean foot placement response for the first step after perturbation served as an indicator of the overall foot placement adjustment in response to a visual disturbance. These measures were previously utilized to study responses to visual perturbations in individuals with cerebral palsy, neurotypical healthy adults, and PwPD (Arcodia et al. 2020; Sansare et al. 2022).

### 2.6. Statistical analysis

Statistical Power

We determined the sample size of fifteen participants based on a priori power analysis in G Power (version 3.1.9.7). We used F-tests module (ANOVA), using a significance level (α = 0.05) and a power of 0.80 to detect a medium-to-large effect size (f = 0.32) due to SR. This effect size was derived from a preliminary data of five responses collected from participants with PD.

Data Analysis

For analyzing the data statistically, we ran a repeated measures ANOVA using SPSS software (version 29), categorizing condition (SR, no-SR) as the within-subject factor. The analysis was carried out separately for each side, the more and less affected sides. The descriptive statistics of baseline data within our participants group were performed using mean and standard deviation (mean ± SD).

## 3. Results

Out of the fifteen participants, only fourteen completed the study under visual perturbation, and no major incidents were reported. One participant was unable to complete the study due to an extreme disturbance by the visual stimuli, causing noticeable drifts off the treadmill. All participants were on medication. The demographic variables for the fourteen participants are presented in Table 2, and the descriptive statistics for the outcome measure are reported in Table 3.

**Table 2.** Mean and standard deviation for the participants demographic data.

	Mean ± SD (*n* = 14)
Age (years)	63.8 ± 6.2
Height (m)	1.75 ± 0.08
Weight (kg)	75.6 ± 10.6
BMI	24.8 ± 3.3
Hoehn & Yahr (# of subjects (stage))	2(0), 6(1), 4(2), 2(3)
UPDRS IA	1.86 ± 3.9
UPDRS IB	6.43 ± 3.2
UPDRS II	9.21 ± 6.1
UPDRS III	20.5 ± 13.5
UPDRS IV	3.14 ± 3.2

Permission to use the MDS-UPDRS rating scale in this study was granted from MDS.

**Table 3.** Descriptive statistics for the outcome measures.

Outcome Measure		df	F value	*p* value	Partial Eta Squared
AUC ML CoM excursion (cm.sec)	More Affected Side	(1,13)	2.558	0.134	0.164
	Less Affected Side	(1,13)	0.231	0.639	0.017
Peak ML CoM excursion (cm)	More Affected Side	(1,13)	6.205	0.027	0.323
	Less Affected Side	(1,13)	0.110	0.745	0.008
Peak time (sec)	More Affected Side	(1,13)	0.003	0.956	0.000
	Less Affected Side	(1,13)	0.211	0.654	0.016
Subtalar angle (deg.sec)	More Affected Side	(1,13)	0.151	0.704	0.011
	Less Affected Side	(1,13)	0.234	0.637	0.018
Foot placement (cm)	More Affected Side	(1,13)	1.490	0.244	0.103
	Less Affected Side	(1,13)	3.771	0.074	0.225

### 3.1. CoM response

**Figure 3.**
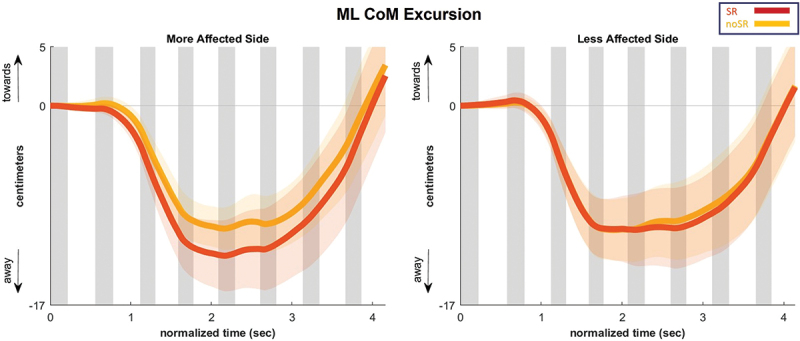
Ml CoM excursion as an average for all the participants for the eight steps following a fall stimulus, showing both stimulation conditions (SR and no-SR) for the more affected (left panel) and less affected (right panel) sides. The gray lines represent double-stance phase, the white areas represent single-stance. The shaded regions surrounding each trajectory indicate the 95% confidence interval and each step normalized to 100 timepoints. Abbreviations: CoM = center of mass; ML = medio-lateral; SR = stochastic resonance.

**Figure 4.**
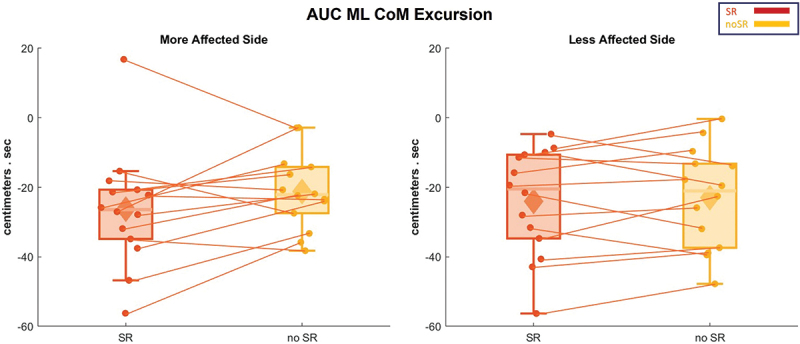
The AUC of the ML CoM excursion for all the participants is represented in box and whisker plots for both conditions (SR and no-SR) at both, more affected (left panel) and less affected (right panel) sides. The solid diamonds representing the mean and the scattered dots representing individual data points. Abbreviations: CoM = center of mass; AUC = area under the curve; ML = medio-lateral; SR = stochastic resonance.

The average CoM displacement in the ML direction is presented in Figure 3, and its AUC is shown as box and whisker plots in Figure 4. Highlighting the AUC of ML CoM displacements, there were no significant effects for condition, SR compared to no-SR, in the more and less affected sides (*p* = 0.134 and *p* = 0.639).

**Figure 5.**
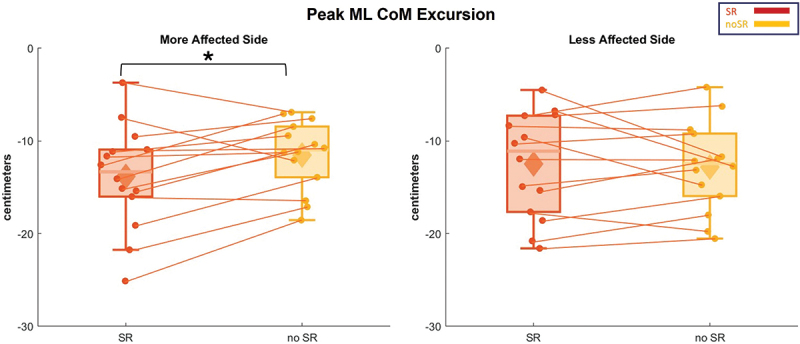
The peak ML CoM excursion for all the participants is represented in box and whisker plots for both conditions (SR and no-SR) at both, more affected (left panel) and less affected (right panel) sides. The solid diamonds representing the mean and the scattered dots representing individual data points. Asterisk indicates significant value of *p* < 0.05. Abbreviations: CoM = center of mass; ML = medio-lateral; SR = stochastic resonance.

Figure 5 illustrates the box and whisker plots for the peak of CoM displacement on both sides. In the analysis of the more affected side, a significant effect for condition was detected in the peak of CoM excursion (*p* = 0.027), which was higher in the SR condition. This might indicate an increase in the body sway with the SR condition in the more affected side. In contrast, in the less affected side, the participants showed no statistical difference in response between the SR and no-SR conditions (*p* = 0.745).

**Figure 6.**
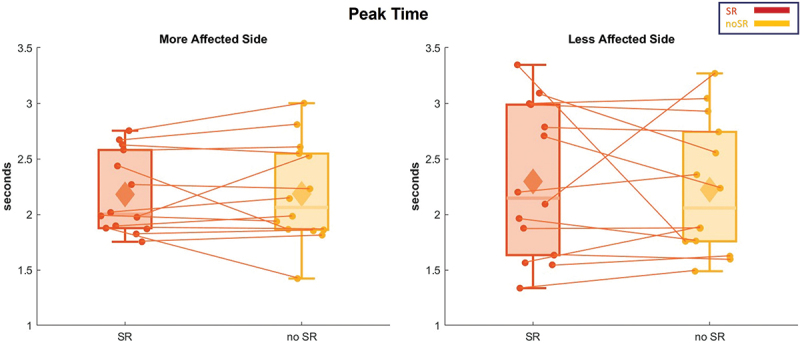
The peak time of the ML CoM excursion for all the participants is represented in box and whisker plots for both conditions (SR and no-SR) at both, more affected (left panel) and less affected (right panel) sides. The solid diamonds representing the mean and the scattered dots representing individual data points. Abbreviations: CoM = center of mass; ML = medio-lateral; SR = stochastic resonance.

For the peak time of CoM displacement, Figure 6 illustrates that there was no significant effect of SR stimulation in delaying or advancing the peak of CoM excursion in the more and less affected sides (*p* = 0.956 and 0.654, respectively).

### 3.2. Balance mechanisms

**Figure 7.**
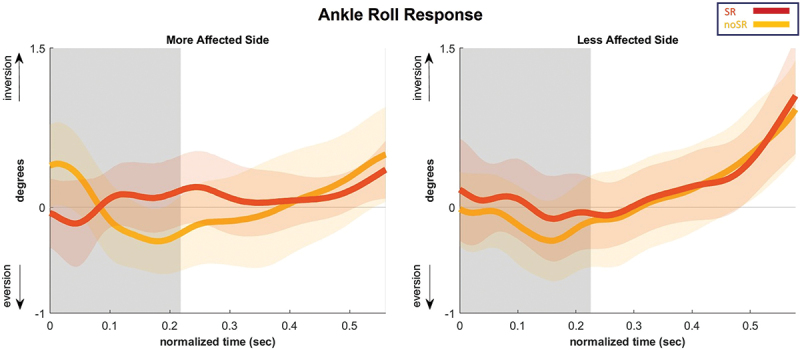
Subtalar angle as an average for all the participants for the first step following a fall stimulus, showing both stimulation conditions (SR and no-SR) for the more affected (left panel) and less affected (right panel) sides. The gray lines represent double-stance phase, the white areas represent single-stance. Positive Y-axis indicates inversion while the negative indicates eversion. The shaded regions surrounding each trajectory indicate the 95% confidence interval and each step normalized to 100 timepoints. Abbreviations: SR = stochastic resonance.

**Figure 8.**
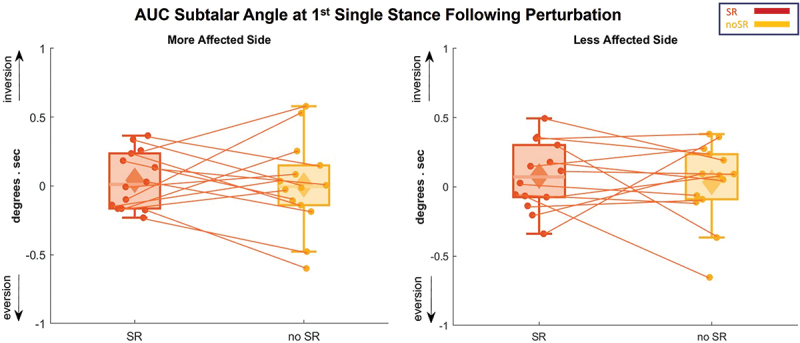
The AUC for subtalar angle is represented in box and whisker plots for all the participants for both conditions (SR and no-SR) at more affected (left panel) and less affected (right panel) sides. The solid diamonds representing the mean and the scattered dots representing individual data points. Abbreviations: AUC = area under the curve; SR = stochastic resonance.

The ankle roll response is presented in Figure 7, which illustrates the average subtalar joint angle for the first step following the perturbation. Figure 8 presents the subtalar angle variations using box and whisker plots. Our findings could not detect any significant condition effect on the AUC for the subtalar angle on both sides, with a *p*-value of 0.704 and 0.637 for the more and less affected sides, respectively.

**Figure 9.**
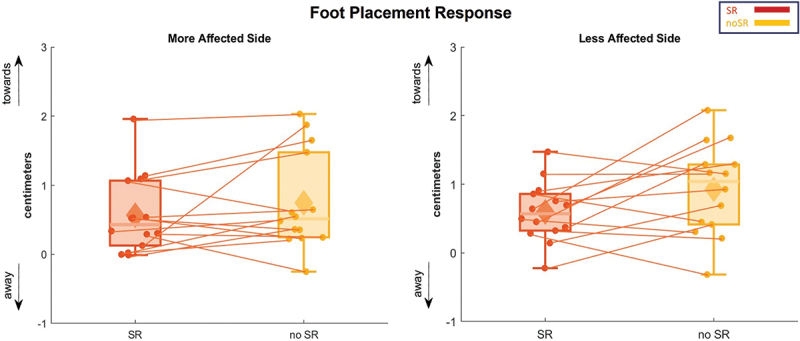
The foot placement response for the first step following visual perturbation is represented in box and whisker plots for both conditions (SR and no-SR) at more affected (left panel) and less affected (right panel) sides. The solid diamonds representing the mean and the scattered dots representing individual data points. Abbreviations: SR = stochastic resonance.

Figure 9 depicts the average foot placement for the first step after perturbation. Similar to the ankle balance mechanism, the analysis of the foot placement revealed no significant changes attributable to the condition on the affected (*p* = 0.224) and the less affected side (*p* = 0.074).

## 4. Discussion

We investigated immediate effects of SR stimulation on dynamic balance control in PwPD during visually perturbing environments via virtual reality. Our hypotheses of SR stimulation reducing the CoM response to visual perturbation and enhancing distal balance mechanisms over proximal ones were not supported. SR stimulation resulted in larger CoM peak excursion compared to no-SR for what we defined as the more affected side. Two potential interpretations for these findings could be: 1) overdosing the applied electric stimulation and 2) misidentifying the more affected side. These errors could emphasize visual dependence for balance control in the PD population (Arcodia 2024), which SR was unable to reduce through enhanced distal sensory inputs.

Our results diverge from other studies conducted in different pathological cohorts. Sansare’s study demonstrated enhanced balance in children with cerebral palsy, whereas Zandiyeh’s work reported improved proprioception in patients after ACL reconstruction (Zandiyeh et al. 2019; Sansare A, Arcodia M, et al. 2024). The varying results may indicate the diverse characteristics of sensory deficiencies in different conditions and the need to align intervention intensity with specific deficit profiles. In contrast to groups exhibiting distinct unilateral deficits, individuals with early-stage Parkinson disease may experience nuanced, bilateral sensory alterations that react differently to sensory reinforcement augmentation.

We identified the more affected side based on motor symptoms confirmed by MDS-UPDRS-III results. Since SR optimal intensity is selected based on the more affected side and applied to both lower extremities, accuracy in lateralization selection is crucial. When assessing motor signs, unilateral upper extremity tremors were prominent in our cohort, making these tremors the main source for lateralization selection. However, lower extremity tremors might evolve at disease onset or progression and appear on the same or opposite side to upper extremity tremors (Pasquini et al. 2018; Dirkx and Bologna 2022; Seuthe et al. 2024). This heterogeneity was observed in one participant with right lower extremity tremors and left upper extremity tremors. Since stability during walking relies heavily on lower extremities, determining the more affected side based on lower limb tremors was reasonable in this case. By doing so, we found noticeable reduction in medio-lateral sway and better use of ankle strategy with SR. A recent study found that asymmetry across gait and motor symptoms severity in PwPD did not correlate, highlighting mismatches in side and degree of asymmetry across motor domains (Seuthe et al. 2024).

Due to disease heterogeneity and complexity, some PwPD do not present with motor symptoms in early phases, weakening ability to identify a more affected side based on motor signs (Cardoso et al. 2024). Prior work showed that SR improvement in children with cerebral palsy related to baseline sensory deficits (Sansare et al. 2025). Selecting the more affected side based on sensory deficits might yield greater improvements with SR, given the sensory system’s role in gait and postural control. Unlike the laterality mismatch in PD signs, people with knee ligament injuries and cerebral palsy, who benefited from SR stimulation, have clearer affected sides, suggesting that accurate side selection might impact SR effects on dynamic stability.

Although increased CoM excursion generally indicates decreasing stability, different interpretations warrant examination. The large excursions with SR may indicate improved sensory feedback, facilitating a more precise understanding of body position and resulting in more suitable corrective actions instead of inflexible, overly cautious movements. Furthermore, augmented confidence from heightened sensory information may enable individuals to utilize their complete range of motion more efficiently, manifesting as increased sway but perhaps indicating superior adaptive capacity. The lack of equivalent adjustments in the lower extremity mechanisms, including ankle roll and foot placement, suggests that the large CoM excursion is more likely indicative of sensory disruption rather than enhanced adaptive responses.

Although sensory deficits in PwPD were reported (Kelemen et al. 2022; Corrà et al. 2023), there is no definite consensus on type, pathophysiology, or timing of these impairments. Since SR stimulation theoretically augments proprioceptive inputs (Zandiyeh et al. 2019; Chen et al. 2020; Severini et al. 2023), individuals with early-stage PD may receive extra proprioceptive inputs, potentially leading to more noise in their somatosensory system and disrupting sensory integration. Proper sensory integration through reweighting is crucial for dynamic stability, where individuals upweight inputs from reliable sources while downweighing unreliable ones (Oie et al. 2002; Peterka 2002).

Recent work showed reduced ankle responses in PD during visual disturbances (Arcodia et al. 2020). Regarding balance mechanisms, our findings were inconclusive. We might justify insignificant results by proprioceptive and vibrotactile sensory impairments not greatly manifested at early phases, as observed in our somatosensory testing where vibration sense was most impaired. Alternatively, unexplored contributors to CoM excursion changes might include alterations in cortical networks for postural control. Early-stage PwPD may have proper sensory integration abilities but poor processing rates (Cooper et al. 1994; Sawamoto et al. 2002; Vriend et al. 2020; Arroyo et al. 2021), causing delayed functional stability after perturbations.

Previous studies showed promising results using SR with populations having sensory impairments. A systematic review found whole-body vibration based on SR improved balance in PD (Sharififar et al. 2014). Similar findings were reported when whole-body-vibration in stochastic pattern enhanced postural stability in PwPD (Kaut et al. 2016). Our inconsistent findings may relate to methodological differences, including using whole-body-vibration directly to feet insoles in referenced studies versus vestibular system application in others (Samoudi et al. 2015). Another difference is our walking protocol in virtual reality versus standing protocols in previous research.

These findings have important implications for future SR interventions in PwPD. The presence of kinesiophobia in 77.3% of PD participants (Jiménez-Cebrián et al. 2021) and demonstrated impacts on quality of life domains including physical activity and social capacity (Navarro-Flores et al. 2022) suggest that SR interventions must consider the broader biopsychosocial context. Rather than a one-size-fits-all approach, personalized sensory assessment protocols could guide SR parameter selection and identify optimal candidates for this intervention. Clinicians should consider that SR may not be universally beneficial and could potentially worsen stability in some individuals, particularly those with intact or mildly impaired sensory systems who may already be dealing with movement-related fears.

### 4.1. Limitations

Limitations include inclusion criteria of PwPD within H&Y stages ≤ III, with most participants at stages I or II. At these early stages, sensory deficits may not yet affect functionality compared to advanced stages. Additionally, our physically active, regularly exercising participants may not represent the general PD population, as well as emphasizing the positive influence exercise has on physical performance (Hvingelby et al. 2022; Qian et al. 2023; Lorenzo-García et al. 2024; Mak et al. 2024). Participants were also ON medication, enhancing physical performance by alleviating motor symptoms (Dibble et al. 2015). In terms of side selection, future research should incorporate multi-modal approaches for determining the more affected side, encompassing quantitative sensory testing (vibration thresholds and joint position sense), asymmetry in postural responses, and biomechanical evaluations of gait parameters. This thorough methodology may more effectively discern the side most predisposed to gain from sensory enhancement by SR stimulation. Moreover, integrating thorough sensory assessments, such as tactile discrimination and proprioceptive acuity, may inform personalized SR parameter selection and anticipate treatment efficacy.

## Conclusion

This study showed that personalized SR stimulation increased body sway on the more affected side in early-stage PwPD without enhancing balance mechanisms, indicating that SR may generate sensory noise rather than improvement in this group. These findings underscore the significance of meticulous sensory profiling and identification of the more affected side when developing sensory rehabilitation therapies for individuals with Parkinson disease. The findings challenge the presumption that sensory augmentation is uniformly advantageous and emphasize the need for tailored strategies based on individual deficit profiles. Future studies should focus on finding optimal candidates and parameters for SR stimulation by thorough sensory evaluation, while also considering illness stage and severity during the implementation of SR therapies.

## Data Availability

The data supporting the findings of this study are available on request from the corresponding author. The data are not publicly available due to privacy or ethical restrictions.
